# Risk of Adverse Outcomes in Females Taking Oral Creatine Monohydrate: A Systematic Review and Meta-Analysis

**DOI:** 10.3390/nu12061780

**Published:** 2020-06-15

**Authors:** Deborah L. de Guingand, Kirsten R. Palmer, Rodney J. Snow, Miranda L. Davies-Tuck, Stacey J. Ellery

**Affiliations:** 1 The Ritchie Centre, Hudson Institute of Medical Research, Melbourne 3168, Australia; miranda.tuck@hudson.org.au (M.L.D.-T.); stacey.ellery@hudson.org.au (S.J.E.); 2 Department of Obstetrics and Gynaecology, Monash University, Melbourne 3168, Australia; kirsten.palmer@monash.edu; 3 Monash Health, Monash Medical Centre, Melbourne 3168, Australia; 4 Institute of Physical Activity and Nutrition, Deakin University, Melbourne 3125, Australia; rod.snow@deakin.edu.au

**Keywords:** creatine monohydrate, supplementation, adverse outcomes, safety, human, female

## Abstract

Creatine Monohydrate (CrM) is a dietary supplement routinely used as an ergogenic aid for sport and training, and as a potential therapeutic aid to augment different disease processes. Despite its increased use in recent years, studies reporting potential adverse outcomes of CrM have been mostly derived from male or mixed sex populations. A systematic search was conducted, which included female participants on CrM, where adverse outcomes were reported, with meta-analysis performed where appropriate. Six hundred and fifty-six studies were identified where creatine supplementation was the primary intervention; fifty-eight were female only studies (9%). Twenty-nine studies monitored for adverse outcomes, with 951 participants. There were no deaths or serious adverse outcomes reported. There were no significant differences in total adverse events, (risk ratio (RR) 1.24 (95% CI 0.51, 2.98)), gastrointestinal events, (RR 1.09 (95% CI 0.53, 2.24)), or weight gain, (mean difference (MD) 1.24 kg pre-intervention, (95% CI −0.34, 2.82)) to 1.37 kg post-intervention (95% CI −0.50, 3.23)), in CrM supplemented females, when stratified by dosing regimen and subject to meta-analysis. No statistically significant difference was reported in measures of renal or hepatic function. In conclusion, mortality and serious adverse events are not associated with CrM supplementation in females. Nor does the use of creatine supplementation increase the risk of total adverse outcomes, weight gain or renal and hepatic complications in females. However, all future studies of creatine supplementation in females should consider surveillance and comprehensive reporting of adverse outcomes to better inform participants and health professionals involved in future trials.

## 1. Introduction

The creatine kinase circuit is integral to cellular bioenergetics and maintenance of ATP production, especially in skeletal muscle [1,2]. This circuit is also essential for normal neurodevelopment and cognitive function [3,4,5]. Creatine monohydrate (CrM) has been used as a dietary supplement to enhance sporting performance, and as a potential therapeutic aid to modify neurodegenerative disease or musculo-skeletal disorders, such as Huntington’s disease [6], amyotrophic lateral sclerosis [7,8], and certain muscular dystrophies [9]. These studies have shown mixed results; from some clinical benefits in muscular dystrophies, to no apparent clinical improvement in neurodegenerative diseases [10,11]. Creatine supplementation became popularised as an ergogenic aid after it was first reported to increase the total creatine content in the skeletal muscle of men [12]. The first therapeutic human trial with CrM was undertaken to determine if it could mitigate the deterioration in gyrate atrophy [13]. After several decades of efficacy studies, in 2017, The International Society of Sports Nutrition (ISSN) concluded that creatine is likely to be more beneficial than harmful to many populations [14].

While there have been more than eighty reviews reporting the ergogenic or therapeutic effect of creatine supplementation, only thirty-four reviews have addressed the safety of this supplement. Some of these reviews have been highly specific, targeting particular areas of interest [15,16,17,18], but none have been sex specific. There have only been two systematic reviews to date, one which focused on renal function [19] and the other on fluid dynamics [20] both were conducted in mixed sex populations. The first general safety reviews were conducted in 1998–2000 [21,22,23,24] and more recently in 2011–2012 [25,26,27]. The heterogeneity of reviews can make it difficult to assess the safety of creatine supplementation relative to population characteristics, such as sex. Study design, methodology and dosing regimens may also potentially modify adverse outcome reporting.

To the best of our knowledge there has not been a systematic review on the safety of creatine supplementation in a female only population. Indeed, a paucity of current safety data exists in females. This is despite estimates that up to 65% of female athletes use ergogenic aids (including CrM, caffeine and beta-alanine) [28] and despite females being over-represented in some disease processes, such as auto-immune disorders [29,30,31], and major depressive disorders (MDD) [32,33,34,35,36] in which creatine has been trialled as a therapeutic intervention. Pre-clinical animal studies show a potential benefit in supplementing a pregnancy with creatine to improve neonatal outcomes [37,38,39,40,41] and studies have now been conducted to characterise creatine metabolism in human pregnancy [42]. Combined, these ergogenic practices and disease processes, which can occur during a female’s reproductive years, make understanding the safety of creatine supplementation in this population important and relevant [43]. The primary purpose of this systematic review is to report the number of adverse outcomes (both events and effects) in females taking oral creatine supplementation. This systematic review aims to consolidate current evidence, from both ergogenic studies and therapeutic trials, to accurately inform on the safety of creatine supplementation in females.

## 2. Methods

This review has been registered with PROSPERO (access ID CRD42018105999) and undertaken in keeping with Preferred Reporting Items for Systematic Reviews and Meta-Analyses (PRISMA) guidelines [44].

### 2.1. Study Selection

We reviewed any trial or case reports where CrM was used in a female population for any purpose, and which included explicit reporting or monitoring of adverse outcomes, including changes in body weight. Studies included randomised controlled trials, open-label trials, dose-escalating trials, crossover studies, repeated measures trials with parallel arms, case studies, and pharmacokinetic and pharmacodynamic studies. We included studies of females from the age of menarche onwards, who were given oral CrM, including trials where concomitant medication was used, when reported evenly across groups. Studies were excluded if undertaken in paediatric or mixed sex populations, and populations with known creatine deficiency syndromes or mitochondrial disorders. All included studies had institutional human ethics approval.

### 2.2. Search Strategy

We searched the following databases from inception to October 2019, with auto search alerts continuing up to submission date. This included all languages and grey literature [45]. Databases searched included Medline (Ovid), Embase (Elsevier), Web of Science (Clarivate Analytics), Scopus (Elsevier), SPORTDiscus, ScienceDirect, CINAHL and the Cochrane Database of clinical trials and systematic reviews. All databases were searched broadly, using the keywords, ‘creatine’, ‘creatine phosphate’, ‘total creatine’, ‘phosphocreatine’ and ‘phosphorylcreatine’, and then combined with a search of all ‘creatine supplementation’, ‘creatine monohydrate’, ‘oral creatine’, and its known derivations (File S1 A). Medline and Embase search engines were able to be further refined, with a narrower search of a priori known adverse events (e.g., creatine AND nausea), and exclusion of animal and creatine deficiency studies (File S1 B). Both strategies were applied and compared, to validate the search strategies and enhance the sensitivity and precision of the search [46,47,48]. Only the broadest search strategy was able to be applied in all other databases (All creatine AND All creatine supplementation) in order to capture all adverse outcomes. A manual search of references in previous safety reviews was also undertaken. Attempts were made to contact trial authors where contact details were provided, to clarify information such as the population mix, specific intervention(s) used, or to request original laboratory data. In cases of no reply, where the message was undeliverable, or the author contact details could not be located, no subsequent attempt was made.

### 2.3. Data Extraction

The lead author conducted searches, screened titles and abstracts, obtained full manuscripts and was responsible for extracting and collating the relevant raw data into a spreadsheet. No a priori assumptions were made about which adverse outcomes to collect and report. Where studies reported a wide range of potential adverse events and effects, these were discussed by three authors and where consensus was reached, symptoms were attributed to the appropriate organ system. We subsequently excluded studies that reported specifically on changes to other metabolic pathways in response to creatine supplementation, as differentiating these outcomes into positive or negative effects was beyond the scope of this review [49,50,51,52,53,54,55]. Where included ergogenic studies reported changes in lactate, heart rate, blood pH, VO_2_ Max or insulin resistance, we could not clearly define these as adverse outcomes to creatine supplementation, rather, we determined them more likely associated with a physiological response to concurrent exercise regimens.

The primary outcome of this review was to report deaths, serious adverse events (any outcome that causes life-threatening events; requirement for hospitalisation or prolongation of existing hospitalisation; persistent or significant disability; or any events that are considered medically important), adverse events (any adverse outcome reported by the participant as a symptom that occurred while a person was taking an intervention at any dose, but the event was not, or has not been assessed as necessarily attributable to the intervention taken), adverse effects (any unwanted outcomes of which the person was not aware; usually detected by laboratory tests or by clinical investigations) in any organ systems, and weight gain with CrM supplementation. Body composition changes were measured in a variety of ways within studies; however, we determined overall weight gain as the only body composition change that could be viewed as a potential adverse effect in females, especially those not taking creatine for sporting improvement. Secondary outcomes were to describe factors that may modify adverse outcome reporting such as study design, methodology, reporting bias and dosing regimens.

### 2.4. Data Analysis and Statistical Methods

Characteristics of the included studies were tabulated. Studies were subsequently categorised as ergogenic, (assessing outcomes related to muscle strength, muscle power, and functional performance), or therapeutic, (assessing outcomes related to improvement in cognitive function or modification of disease processes). We defined pre-menopausal females as women over the age of menarche, determined as 13 years of age or above, and less than 50 years of age [56]. Where it was clear how adverse outcomes were collected and measured we categorised these as participant-initiated reporting, researcher-initiated reporting, or unknown. Studies were characterised by dosing regimens, where participants received either a maintenance dose or a loading dose only, or they received a combined dose; which included a loading dose followed by a maintenance dose. Studies reported adverse outcomes in one of three ways. Where an adverse outcome was reported as a single episode per participant across groups, these were reported as a dichotomous variable (YES/NO) and subject to meta-analysis using the Cochrane RevMan 5.3 software(Copenhagen, Denmark) and stratified by dosing regimen. Single participant outcomes that could be pooled were presented in the meta-analysis as a risk ratio (RR) applying a random effects model with accompanying 95% confidence intervals (CI’s) using the Cochrane RevMan 5.3 software and stratified by dosing regimen [57]. Studies that reported differently, by either counting multiple symptoms per participant across groups, or by describing zero events across groups, are shown in the meta-analysis, but do not contribute data to the overall effect estimate. The gastrointestinal (GIT) tract was the only system where sufficient numbers of adverse events were clearly attributable to this system and could be subject to meta-analysis. Heterogeneity was measured with the I^2^ statistic. We undertook sensitivity analysis by exposing the data to different methods (fixed vs. random effect) and removing and testing changes in the number of events within studies (where events were minimal, or zero, in one group).

Adverse effects (signs), reported as biochemical laboratory values or measurements, were grouped according to the organ system assessed. Included studies only reported on biomarkers of the renal and/or hepatic system. Reporting of laboratory data was too heterogenous to undertake meta-analysis so where values were presented, these were tabulated as group means, pre- and post-intervention, and the change reported as percentage (%) post-intervention. Where laboratory values were not provided in the manuscript, but authors identified that these systems were monitored, their findings are presented in Table 1. We were unable to ascertain change across all reported body composition parameters (too much heterogeneity) therefore we have presented overall pre- and post-intervention mean group weights, stratified by dosing regimens. These are expressed as a mean difference (MD) in kilograms, applying a random effects model, with accompanying 95% CI’s, and I^2^ statistic. Where studies could not be included in the meta-analysis, we describe their individual findings. Finally, we describe attrition rates and reasons for attrition. Losses are presented as a percentage of the total cohort, and reasons for attrition are reported where provided.

### 2.5. Risk of Bias

We assessed risk of bias across all studies using the Cochrane Handbook for Systematic Reviews of interventions, the GRADE handbook, and ROBINS-1 tool for open label trials [58,59,60]. The overall and individual risk of bias tables were constructed, with studies categorised as high, unclear, or low risk of bias, across seven predefined domains. Two authors assessed each study independently and a third author was consulted if agreement could not be reached.

## 3. Results

### 3.1. Search Characteristics

**Figure 1:**
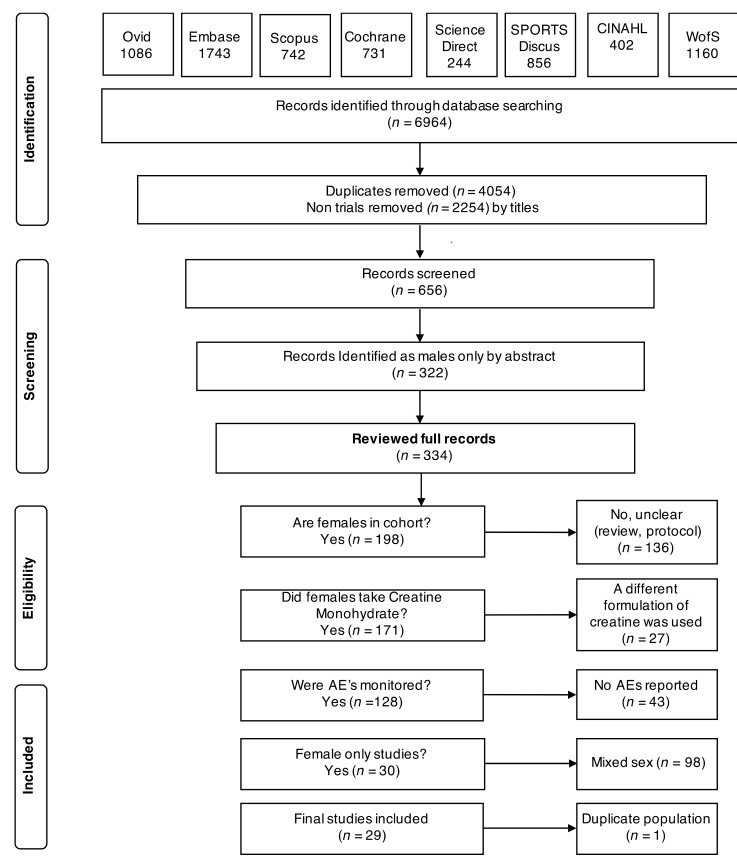
Organisation of article review process as a PRISMA 2009 flow diagram.

Figure 1 outlines the PRISMA flow diagram for retrieving, reviewing, screening and final inclusion of studies. In brief, we retrieved 6964 articles and reviewed 334 full text articles. Twenty-nine articles were included in the final analysis.

### 3.2. Study and Participant Characteristics

Characteristics of studies that met our inclusion criteria are outlined in Table 1. They included three open label trials [34,35,61], a placebo-controlled dose-escalating study [33], two randomised placebo-controlled crossover design studies [62,63], and twenty-three randomised trials with a placebo arm [32,64,65,66,67,68,69,70,71,72,73,74,75,76,77,78,79,80,81,82,83,84,85]. The total number of consented participants was 951 with a sample size between 5 and 149 participants per study, with the median number of participants per study being 24. Eighteen studies were categorised as ergogenic and eleven as therapeutic trials. Studies used different methodology for collection of adverse outcome data. Therapeutic studies were more likely to undertake enhanced reporting of adverse events (via researcher interview, or tool-based surveys or questionnaires), include a data safety committee and be conducted in tertiary care facilities (73%). Ergogenic studies were more likely to use passive methods of data reporting such as participant self-reporting and were mostly conducted in sporting laboratories or facilities (69%). All studies had varied dosing regimens, ranging from 1–30 grams (g) per day, with duration of treatment ranging from 4 days to 365 days. The age range in years in the CrM groups were 16–67, and 16–68 in the placebo groups. Seven studies (24%) contained post-menopausal females and twenty-two studies (76%) included pre-menopausal females.

### 3.3. Risk of Bias

**Figure 2:**
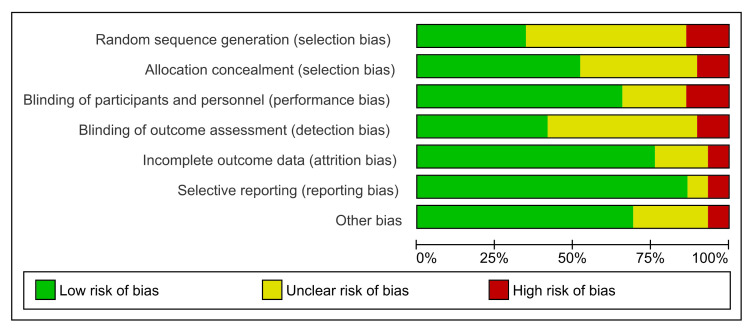
Overall risk of bias for included studies. Data are presented as percentages of risk assessment across 7 predefined domains.

**Figure 3:**
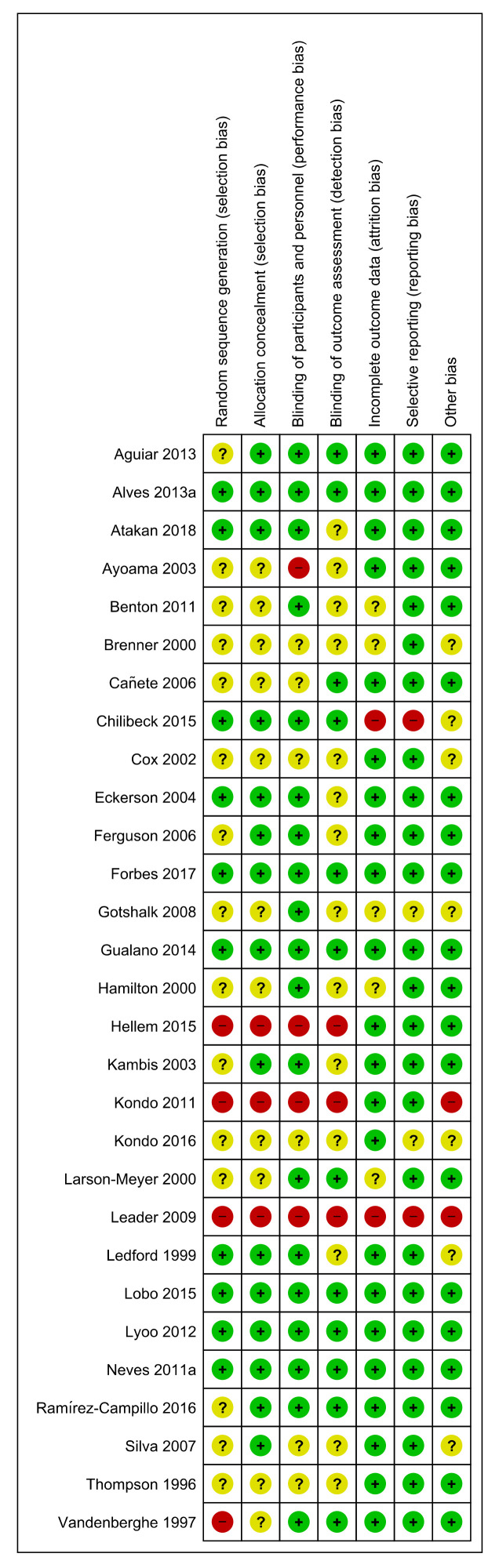
Individual risk of bias for included studies. Data are presented as risk assessment across 7 predefined domains. + (green)=low risk of bias, ? (yellow)=unclear risk of bias, - (red)=high risk of bias

Most studies were well conducted with low risk of bias across the seven defined domains. Low risk ranged from 35–85% across the seven domains. (Figure 2 and Figure 3). High risk of bias was generally low and ranged from 6–12.5% across the seven domains. The domains at lowest risk of bias included selective reporting (85%), incomplete outcome data reporting (75%), blinding of participants and personnel (70%), and risk of other bias (70%). Domains at highest risk of bias included selection bias (12.5%), and performance bias (12%). Poor or unclear reporting of allocation concealment (40%) was evenly shared across ergogenic studies and therapeutic trials. Studies were allocated an unclear risk of bias if the reviewers were unable to determine clearly how the domain bias was addressed. Unclear risk ranged between 6–50% across the seven domains.

### 3.4. Deaths

No deaths were reported in any included study.

### 3.5. Adverse Outcomes (Symptoms and Signs)

Reporting of adverse outcomes varied across the 29 included studies (Table 1 and File S2 A and B). Of the 29 included studies, twenty-four described adverse events, (sometimes referred to as ‘side effects’). One study reported on effects only (signs), describing renal and hepatic markers and body composition, and four studies reported solely on changes in body composition (File S2 C).

Of the twenty-four studies that reported on adverse events, eighteen were included in the meta-analysis. The six studies excluded from the meta-analysis were done so either due to the lack of a comparator group, as was the case for three open label trials [34,35,61], or due to reporting in a manner that prevented appropriate analysis. This included a trial that reported on the percentage within groups that did not experience an adverse event (CrM 74%:Pl 85%), with all events reported as mild (feeling bloated or experiencing a headache) and resolving spontaneously [68], and two studies that reported on illness or injury in participants that resulted in them not completing the study requirements [63,69]. In Ledford et al., one participant was reported as falling ill after repeated exercise with both creatine and placebo supplementation regimens, resulting in an inability to complete outcome measures in this crossover design study [63]. Brenner et al., reported withdrawal of a participant in the CrM group due to compartment syndrome; reporting that symptoms preceded supplementation and were related to injury [69].

**Figure 4:**
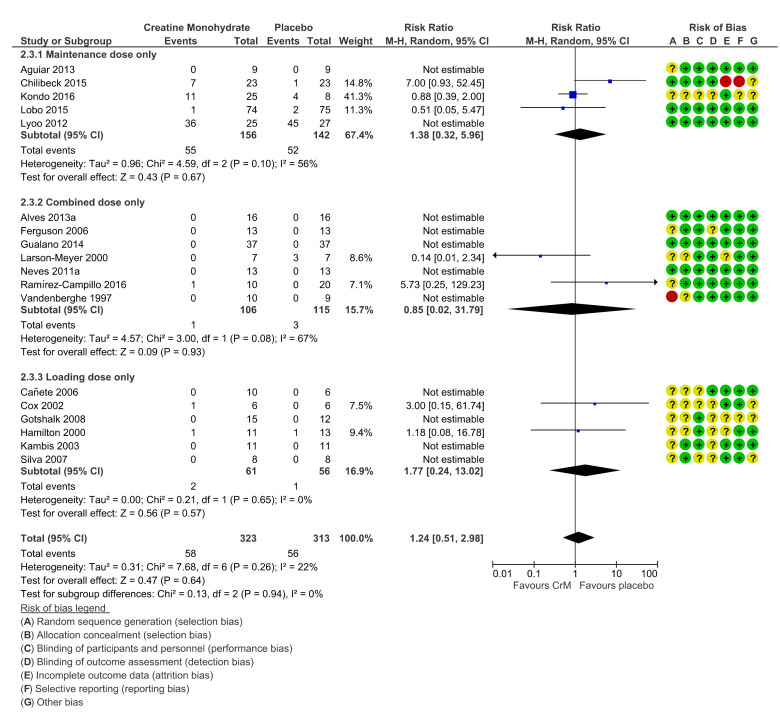
Incidence of adverse events per participant, stratified by dosing regimens. Within Forest Plot, Study or Subgroup = study stratified by dosing regimens; Events = number of adverse events reported; Total = total number of participants in study group; Weight = amount of information contributed by study; M-H = Mantel–Haenszel model; Random= random effects model. Risk of bias; + (green) = low risk of bias, ? (yellow) = unclear risk of bias, - (red) = high risk of bias

Of the 18 studies included in the meta-analysis, ten studies reported zero events (no side effects or adverse events) across either groups [64,65,70,73,75,76,78,81,83,85], and one study counted events differently [32]. Final meta-analysis, stratified by dosing regimens, confirmed no significant differences in adverse events across groups, (RR 1.24 (95% CI 0.51, 2.98); Figure 4). In the majority of cases authors did not speculate on the potential association or causality to CrM, or they determined that events were not attributable to the intervention. Only four therapeutic trials sought to determine potential causality to the intervention [33,34,35,71] and the events ascribed causality to the intervention were GIT disturbances (36%), muscle cramps (2%), and headache (14%).

### 3.6. GIT Events

**Figure 5:**
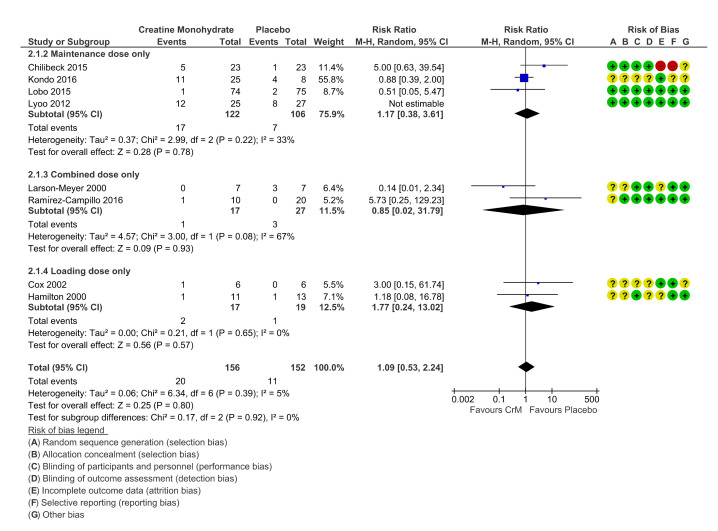
Incidence of gastrointestinal (GIT) events per participant, stratified by dosing regimens. Within Forest Plot, Study or Subgroup = study stratified by dosing regimens; Events = number of adverse events reported; Total = total number of participants in study group; Weight = amount of information contributed by study; M-H = Mantel-Haenszel model; Random=random effects model. Risk of bias; + (green) = low risk of bias, ? (yellow) = unclear risk of bias, - (red)=high risk of bias

The most reported adverse events occurred in the GIT system with eight placebo-controlled studies reporting GIT symptoms, (thirty-two in CrM group, and nineteen in the Placebo group). Symptoms reported include nausea, vomiting, GIT discomfort, diarrhoea, constipation, irritable bowel, bloating, feeling of weight gain, dyspepsia, indigestion, decreased appetite. There was no statistically significant difference across groups for GIT events, when stratified for dosing regimens. (RR 1.09 (95% CI 0.53, 2.24); Figure 5).

Four studies identified GIT symptoms as the reason for cessation of the intervention; however, similar numbers were reported across both groups (five in the CrM group, six in the Placebo group) [32,71,79,80]. Chilibeck et al. was the only study to report a statistically significant difference in adverse events across groups when GIT events were combined with reports of muscle cramping (*p* value < 0.05). However, this study found no statistical difference between groups for the overall number of adverse events reported [71].

### 3.7. Renal System

**Figure 6:**
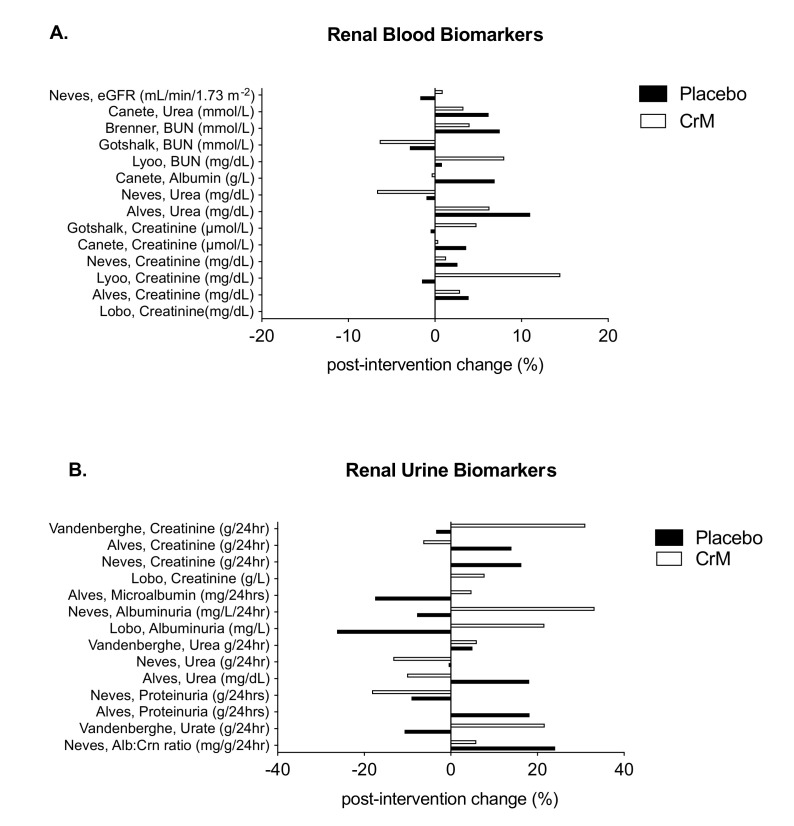
Changes in renal blood biomarkers (**A**) and urine biomarkers (**B**) within placebo (solid bar) and CrM (hatched bar) groups. Data are presented as % change pre-to post-intervention. Units are expressed as presented in the original manuscripts. BUN = blood urea nitrogen, Alb:Crn = albumin:creatinine ratio.

Fourteen studies reported on renal system biomarkers, assessed by either blood or urine, or a combination of both. Eight provided laboratory values by groups, pre-and post-intervention [32,65,69,70,75,80,81,85]. Of the remaining six studies identified, four reported non-significant differences in biomarkers across groups [34,67,71,76], one reported creatinine values as a range across groups pre (0.65–0.85) and post-intervention (0.74–0.88) with no statistical difference seen (*p* =0.16) [33] and the last reported solely on change in serum creatinine levels for the purpose of assessing treatment compliance [35]. Figure 6A represents the studies that reported the mean percentage change in renal blood biomarkers by group, post-intervention, while Figure 6B represents the studies that reported the mean percentage change in renal urine biomarkers by group, post-intervention. Serum creatinine (Crn), was the most assessed renal biomarker (*n* = 9) showing none [80] through to a 14.5% change [32] post-intervention. All levels remained within normal reference ranges and all studies reported no significant differences in serum creatinine between groups over time [32,33,34,65,70,75,76,80,81]. No significant differences were found in blood urea nitrogen (BUN) [32,69,75], plasma urea levels [65,67,70,71,76,81], estimated glomerular filtration rate (eGFR) [81] or creatinine clearance rates (CrnCl) [71]. Chilibeck et al. reported one adverse effect in renal function in the placebo arm of the study, with one participant recording a low creatinine clearance at four and twelve months requiring cessation of placebo, but continuation in the study [71]. Of the four studies that assessed renal urine biomarkers, three noted no significant difference between groups post-intervention [65,80,81], whilst one noted an increase in urinary creatinine in the CrM group, significant only at day one and day three of supplementation in participants receiving a 20 g per day loading dose and not significant at the completion of treatment when participants were on a 5 g per day, maintenance dose [85].

### 3.8. Hepatic System

**Figure 7:**
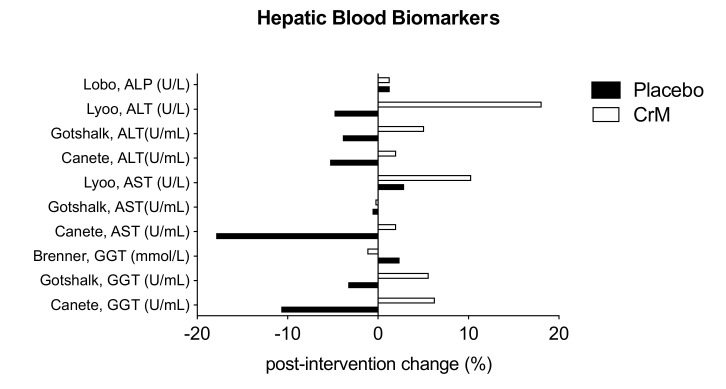
Changes in hepatic blood biomarkers within placebo (solid bar) and CrM (hatched bar) groups. Data are presented as % change pre- to post-intervention. Units are expressed as presented in the original manuscripts. ALP= alkaline phosphatase; AST = aspartate aminotransferase; ALT = alanine aminotransferase; GGT = gamma glutamyl transferase.

Eight studies reported on biomarkers of hepato-biliary function, with all reporting no statistically significant difference across groups in any measured liver enzymes. Five provided laboratory values by groups, pre-and post-intervention [32,69,70,75,80]. Three studies that did not provide values reported non-significant differences in biomarkers across groups [67,71,76]. Figure 7 represents the mean percentage of change in blood biomarkers by group, post-intervention, in these studies. Two studies reported elevation in individual liver biomarkers. Lyoo et al. reported three participants (two in CrM group and one in the placebo group) with mild elevation of liver transaminases above the normal reference range at the end of the eight-week study [32]. Chilibeck et al., reported two adverse effects in the placebo arm of his study; one with an elevated ALP which resolved by study end, and another, an elevated bilirubin level, which remained elevated at the study end. Both women continued in the study, but placebo treatment was discontinued [71].

### 3.9. Body Composition Effects

**Figure 8:**
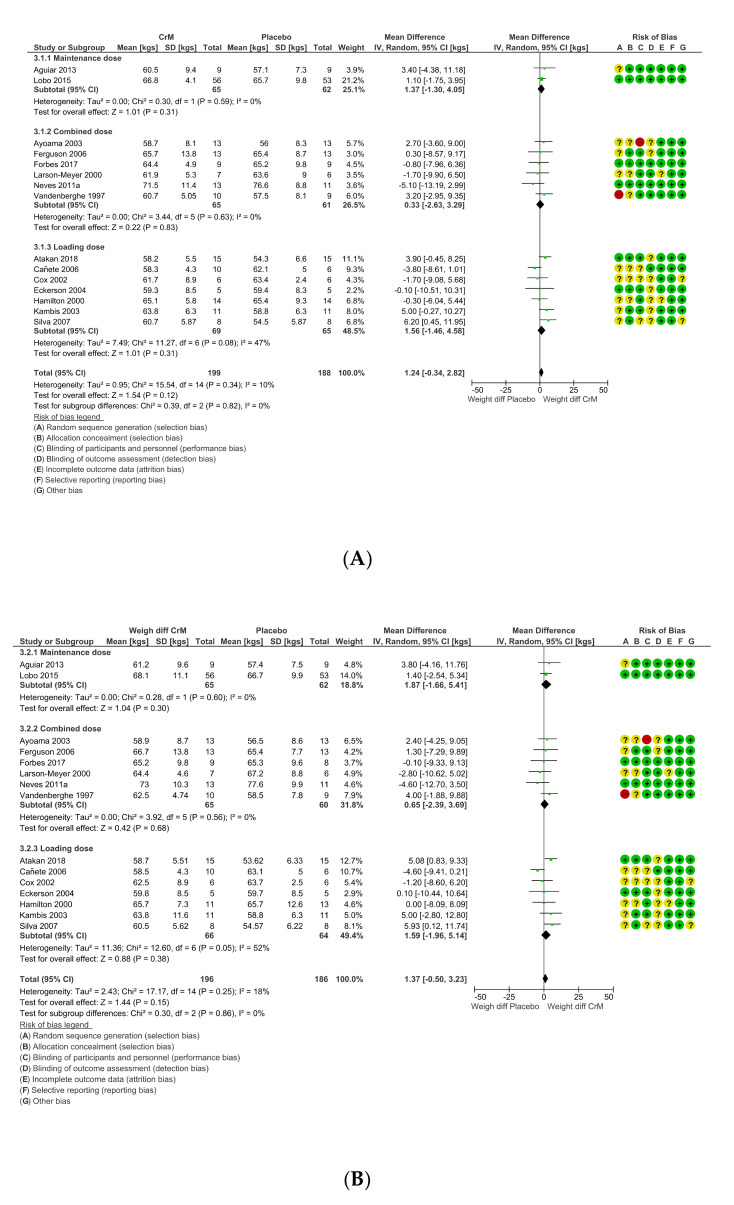
(**A**) Difference in body weights per group, pre-intervention, stratified by dosing regimens. Within Forest Plot, Study or Subgroup = study stratified by dosing regimens; Mean = mean body weight per group in kgs; SD = standard deviation; Total = total number of participants in study group; Weight = amount of information contributed by study; I-V = Inverse Variance random effects model. (**B**) Difference in body weights per group, post-intervention, stratified by dosing regimens. Within Forest Plot, Study or Subgroup = study stratified by dosing regimens; Mean = mean body weight per group in kgs; SD = standard deviation; Total = total number of participants in study group; Weight = amount of information contributed by study; IV = Inverse variance model; Random=random effects model; Risk of bias; + (green) = low risk of bias, ? (yellow) = unclear risk of bias, - (red) = high risk of bias

Twenty-three studies reported on some measure of body composition change, but only fifteen studies provided pre- and post-intervention weight in kgs suitable for meta-analysis. There was no difference in mean weight between groups, pre-intervention (MD 1.24 (95% CI −0.34, 2.82); Figure 8a) to post-intervention (MD 1.37 kg (95% CI −0.50, 3.23); Figure 8b). Studies (*n* = 8) not included in the meta-analysis reported on different parameters of change in body composition. CrM did not change lean body mass over time in young healthy swimmers [84], or body mass (BM) in young healthy females performing repeated cycle ergometer tests [63]. Brenner et al. determined no supplement effect in young healthy lacrosse players, as both groups experienced a 0.5 kg increase in body weight, with a tendency toward a decrease in percentage of body fat (% BF) in the CrM group over time [69]. Kondo et al. reported no significant difference in body weight gains (measured in pounds) across all four groups of adolescents with MDD (three in CrM group and one in the Placebo group) [33]. Gualano and Chilibeck et al. both reported on post-menopausal females undertaking resistance training, with improvement in appendicular lean muscle mass and no change in whole body lean tissue mass over time, respectively [71,76]. Only two studies reported an increase in body mass, Gotshalk et al. reported no change in % BF, but an increase in BM and fat free mass in the CrM group only [75], whilst Ramirez-Campillo et al. attributed a change in body mass index (BMI) of 1.4% (pre-to post-intervention), to CrM supplementation, as the change did not occur in either the placebo or control arm of the study [82]. Studies not reporting on body composition were all therapeutic trials [32,34,35,61,65,68].

### 3.10. A Comment on Cardiovascular Effects or Events

Only two of the 29 included studies reported outcomes in the cardiovascular system. No serious adverse outcomes were reported. In terms of adverse events, one study reported no significant difference in blood pressure across groups after seven days of supplementation, and another reported palpitations in a participant in the placebo arm of the trial [32,75]. Whilst headache was reported in two open label studies and one placebo-controlled study, two of these studies deemed symptoms more likely related to the concomitant medication participants were taking [32,35].

### 3.11. Dosing Regimens

Dosing regimens varied across studies, with three regimens identified; loading dose (*n* = 10 studies; 34.5%) combination regimen (a loading dose followed by a maintenance dose) (*n* = 10 studies; 34.5%), or a maintenance dose only (*n* = 9 studies; 31%). The median loading dose was 20 g per day (range 15–30 g) and the median maintenance dose was 5 g per day (range 1–10 g). For those on loading regimens only (short-term regimen), participants were loaded between 4 and 9 days, (mean of 5 days). Combination regimens used similar loading dose regimens of 5–7 days with the subsequent maintenance regimen varying between 21 and 166 days (mean of 76 days), (medium-term regimen), while those on a maintenance dose remained on intervention for between 42 to 365 days (mean of 126 days; long-term regimen). Dosing regimens did not significantly modify the overall effect of adverse outcome reporting; with all dosing regimens subject to meta-analysis, remaining statistically non-significant.

### 3.12. Withdrawals, Loss to Follow Up, Cessation of Intervention

Sixteen studies reported on attrition; ten were therapeutic trials and six were ergogenic studies. Total attrition rate was low when summed across all studies (14%) but ranged from 1% to 47% in individual studies. Dosing regimens did not affect attrition rates; however, dosing durations and populations studied did. Higher attrition rates (>20%) were reported in five studies, of which four were therapeutic trials. Two continued duration of therapy for one year [71,80] and two had populations with treatment resistant therapy, or who had previous poor treatment compliance [32,61]. While Lyoo et al., found no difference in losses across groups, they did speculate on the higher discontinuation rate in the creatine group in the first two weeks, explaining it could have been due to intolerance, lack of efficacy, or both [32]. The one ergogenic study reporting higher than 20% attrition was conducted over a longer duration of 3 months [79]. The ratio of withdrawal, loss to follow up, or premature cessation of interventions was the same across groups, fifty-three in CrM group and fifty-three in the Placebo group, with a variety of reasons reported for attrition across studies (File S3).

## 4. Discussion

This is the first systematic review to report comprehensively on adverse outcomes in females who ingested CrM. We found no mortality or serious adverse events associated with CrM supplementation in females, which agrees with previous safety reviews in male or mixed sex populations [22,23,27,86,87,88,89,90,91,92].

Whilst deaths have been reported in therapeutic trials of mixed populations taking concurrent CrM, all participants had serious pre-existing comorbidities, or advanced disease processes and none of the deaths were attributable to the creatine intervention [93,94,95,96,97,98,99,100,101,102,103]. Three deaths have previously been reported in males using pre-workout supplements containing creatine; however, no direct causality has ever been established between creatine and these case reports. In all cases males were taking supplements with other ingredients, or other medications, and in some cases had pre-existing comorbidities [104,105,106].

To the best of our knowledge, serious adverse outcomes have only been described in males and only in case reports. These adverse outcomes included renal dysfunction [107,108,109,110,111,112,113,114,115], increased lower leg compartment syndrome [104,116], rhabdomyolysis [116,117,118], ischaemic stroke [119], haemorrhagic stroke [120], liver injury [121,122], atrial fibrillation [123], acute cholestatic liver injury [124] and toxic hepatitis [122]. These events are not replicated in placebo controlled clinical trials [125,126,127,128,129]. Compartment syndrome has been reported in the literature often associated with exertional rhabdomyolysis in males undertaking intensive exercise, and supplementing with creatine, [104,116] but there is no evidence supporting a causal link between these symptoms and creatine supplements [87,130]. The only female in this review who experienced this event had symptoms that preceded the commencement of creatine supplementation, and the symptom was reported as injury related [69]. 

No other serious adverse events have been reported in the female literature and our meta-analysis found no statistically significant difference in adverse events in placebo-controlled trials. This finding is supported by both the Norwegian Scientific Committee for Food Safety [131] and Brudnak et al., who reported that side effects such as GIT symptoms, muscle cramping, and nephritis due to CrM supplementation were not supported when subjected to scrutiny in blinded, placebo-controlled studies, indicating reporting was mostly anecdotal and not evidence based [132].

GIT disturbances reported in male or mixed sex populations have been associated with higher dosing regimens, dosing regimens in excess of those recommended by the manufacturer, or concomitant supplementation regimens [96,133,134]. Our findings support other published literature that found no evidence of a significant effect on GIT symptoms, muscle cramping, or renal and hepatic changes across placebo-controlled trials [15,19,90,94,134,135,136,137,138]. This included studies in middle to older age male, or mixed sex populations treated with creatine [94,139,140,141,142,143] and in younger mixed sex populations [25,135,144].

Serum creatinine remains the most commonly used biomarker of renal function and this corresponds to our review. Previous studies have shown no significant change in serum creatinine levels [94,135,144,145,146,147,148,149], or a mild to modest change of up to 13% with creatine supplementation [150,151,152], which correlates with the ranges reported in our studies. Serum creatinine remained within normal range across all studies over the time of supplementation, and this finding corresponds with a previous review that found 91% of studies reported no change or change that remained within normal limits [87]. The potential for change in serum creatinine is considered a normal physiological response to creatine supplementation due to the spontaneous conversion of creatine to creatinine, [2] but like others, we would caution against the use of a single biomarker to interpret a change in renal function. [15,35,87,153,154].

This review found no evidence that CrM supplementation negatively impacts liver function in females, which corresponds to the findings of other studies and reviews [13,138,145,149,152,155,156,157]. The reported mild elevations across two studies in our review, although not explained by the authors, may be explained by the individual characteristics and potential comorbidities of the study populations, and adjunct treatments or therapies [32,71]. The high intra-individual variability in common liver function tests, make interpretation of one-off changes in liver function difficult, and caution should be applied, with interpretation of the full clinical picture [158,159]. Creatine supplementation appears to have no effect on blood pressure; however, this finding is based on limited data and should be viewed with caution. There have been no systematic reviews on blood pressure changes or adverse cardiovascular effects of creatine supplementation in relatively healthy male or mix-sex populations. However, our finding is consistent with no reported blood pressure effect in three published male only placebo-controlled trials with creatine [160,161,162] and one mix-sex study [144]. There has been one systematic review on the use of creatine and creatine analogues in hypertension and cardiovascular disease, which also concluded no change in blood pressure with creatine supplementation in myocardial infarction or heart failure trials [163]. Overall, more thorough investigations are required to definitively rule in or out adverse cardiovascular effects of creatine supplementation in both male and female populations.

Weight gain has been the most reported ‘side effect’ of creatine supplementation in the literature for decades but is mostly reported in males. There is a strongly held perception that creatine supplementation may induce a weight change, and this may not be viewed favourably by females, particularly those of reproductive age, female athletes and coaches [62]. Our review found no significant change in body weight in females, which is in line with other studies reporting none to minimal effect on body composition change in females when compared to males [144,164].

Attrition rates can confound reporting of adverse outcomes [46,47]; however, the studies in our review generally showed low attrition and reported their rates, reducing the impact of selective reporting bias. It is likely that higher attrition rates in therapeutic trials are due to the challenging population groups and the length of time required to determine the effect on study outcome. Future therapeutic trials should consider the duration of supplementation to affect the primary outcome, taking into consideration the population characteristics and factor in potential higher attrition rates (≥20%) in their sample size calculations.

## 5. Strengths and Limitations

This is the first systematic review describing the risk of adverse outcomes in females taking CrM supplementation. The studies included were generally well designed with a low overall risk of bias, excellent reporting on attrition rates with limited to, no, missing data. Our tight selection criteria reduced heterogeneity across studies, meaning the outcomes can confidently be applied to non-pregnant females from menarche to post-menopausal age. 

There are some limitations to this review. Like other reviews, we found most studies had small sample sizes and dosing regimens varied, making comparisons challenging [91]. Our review attempted to address this by pooling data and stratifying studies by dosing regimens. Study design and methodology affect how adverse event data is collected and reported, which can contribute to overall bias [46,165,166,167]. Passive surveillance can lead to a potential under-reporting of events [47], but at the same time, over-reporting can occur in placebo-controlled trials where directed questioning may lead to increased reporting of negative effects in placebo groups, known as the nocebo phenomenon [166,168]. It is possible that different methodologies employed (over and under reporting) affected the findings of this review, as could the tools or instruments used to measure change in body weight pre-and post-intervention. [74,169,170].

## 6. Conclusions

This systematic review provides the first comprehensive description of adverse outcomes reported in post-pubertal, non-pregnant females taking CrM providing reassurance that this dietary supplement appears safe at different dosing regimens. Whilst there may be variation in the number of events reported with creatine supplementation, this variation is not statistically significant. In females, CrM does not appear to cause weight gain, nor adversely impact other major organ systems. Our findings support those of previous safety reviews performed in male or mixed sex populations that conclude CrM is a safe dietary supplement when consumed in doses and regimens recommended by manufacturers and current sporting and government safety bodies worldwide. Future studies should consider larger sample sizes in more homogenous groups (sex specific or disease specific only) and incorporate surveillance and mandatory reporting of adverse outcomes into the study design.

## Figures and Tables

**Table 1:** Characteristics of studies included in Systematic Review.

Study Characteristics					Adverse Outcomes			
Author (Year)	Study Design (Study Type)	Number Treated (Cr:Pl)	Population Type (Mean Age ± SD) (Cr:Pl)	Dosing Regimen M, C or L (Total Days Duration)	General Symptoms (R, S, T.)	Renal System	Hepatic System	Body Comp
AGUIAR (2013)	DBRPCT (E)	18 (9:9)	Post-menopausal (64 ± 4:65 ± 6)	M 5 g/day (84)	No AE’s reported (R)	-	-	No effect
ALVES (2013)	DBRPCT (T)	32 (16:16)	Pre-menopausal (48.7 ± 10.1:49 ± 8.4)	C 20 g/day (5) then 5 g/day (107)	No AE’s reported (S)	No difference between pre-and post-intervention	-	-
ATAKAN (2018)	DBRPCT (E)	30 (15:15)	Pre-menopausal (19.8 ± 1.1, all)	L 0.25 g/kg/day (7)	-	-	-	No effect
AYOAMA (2003)	DBRPCPT (E)	26 (13:13)	Pre-menopausal (19.4 ± 0.8:19.3 ± 0.7)	C 20 g/day (7) then 3 g/day (14)	-	No difference between pre-and post-intervention	No difference between pre-and post-intervention	No effect
BENTON (2011)	DBRPCT (T)	121 (61:60)	Pre-menopausal (20.3 ± 2.1, all)	L 20 g/day (5)	AE’s reported across study (R)	-	-	-
BRENNER (2000)	DBRPCT (E)	20 (10:10)	Pre-menopausal (18.1 ± 7.6:19.5 ± 8.5)	C 20 g/day, (7) then 2 g/day (28)	One AE reported (R)	No difference between pre-and post-intervention	No difference between pre-and post-intervention	No effect
CANETE (2006)	SBRPCT (E)	16 (10:6)	Post-menopausal (67 ± 6:68 ± 4)	L 0.3 g/kg/day (7)	No AE’s reported (S)	No difference between pre-and post-intervention	No difference between pre-and post-intervention	No effect
CHILIBECK (2015)	DBRPCPT (T)	47 (23:24)	Post-menopausal (57 ± 4:57 ± 7)	M 0.1 g/kg/day (365)	AE’s reported across study (R)	No difference between pre-and post-intervention	No difference between pre-and post-intervention	No effect
COX (2002)	DBPCMT (E)	12 (6:6)	Pre-menopausal (22.1 ± 5.4, all)	L 20 g/day (6)	AE’s reported across study (S)	-	-	Effect
ECKERSON (2006)	DBRPCMXT (E)	10 (All)	Pre-menopausal (22 ± 5, all)	L 20 g/day (5)	-	-	-	No effect
FERGUSON (2006)	DBRPCMT (E)	26 (13:13)	Pre-menopausal (24.6 ± 3.4, all)	C 0.3 g/kg/day (7) then 0.03 g/kg/day (63)	No AE’s reported (S)	-	-	No effect
FORBES (2017)	DBRPCT (E)	18 (9:8)	Pre-menopausal (23.8 ± 4.7:22.4 ± 3)	C 0.3 g/kg/day (5) then 0.1 g/kg/day (28)	-	-	-	No effect
GOTSHALK (2008)	DBRPCMT (E)	30 (15:12)	Post-menopausal (63.3 ± 4.6:63 ± 3.8)	L 0.3 g/kg/day (7)	No AE’s reported (S)	No difference between pre-and post-intervention	No difference between pre-and post-intervention	Effect
GUALANO (2014)	DBRPCPT (T)	74 (37:37)	Post-menopausal (66.1 ± 4.8:66.3 ± 6)	C 20 g/day (5) then 5 g/day (161)	No AE’s reported (S)	No difference between pre-and post-intervention	No difference between pre-and post-intervention	Effect
HAMILTON (2000)	SBRCMT (E)	28 (11:13)	Pre-menopausal (22.5 ± 4.23:23.9 ± 4.76)	L 30 g/day (9)	AE’s reported across study (S)	-	-	No effect
HELLEM (2015)	PSOL (T)	14 (All)	Pre-menopausal (37.4 ± 9.9)	M 5 g/day (56)	AE’s reported across study (R)	Increase in serum creatinine reported		-
KAMBIS (2003)	DBRPCMT (E)	22 (11:11)	Pre-menopausal (63.2 ± 6.68:63 ± 6.08)	L 20 g/day (5)	No AE’s reported (S)	-	-	No effect
KONDO (2011)	PSOL (T)	5 (All)	Pre-menopausal 14–18)	M 4 g/day (56)	AE’s reported across study (R)	No abnormal levels reported		-
KONDO * (2016)	DBRPCDRT (T)	33 (25:8)	Pre-menopausal (13–20)	M 2, 4 or 10 g/day (56)	AE’s reported across study (R)	No difference between pre-and post-intervention	-	No effect
LARSON-MEYER (2000)	DBRPCT (E)	14 (7:7)	Pre-menopausal (19 ± 1.5:19.3 ± 1.4)	C 15 g/day (5) then 5 g/day (86)	AE’s reported across study (R)	-	-	No effect
LEADER (2009)	OLT (T)	30 (All)	Pre-menopausal (over 18)	M 3 g/day (7) then 5 g/day (49)	No AE’s reported (S)	-		-
LEDFORD (1999)	DBRPCMXT (E)	10 (All)	Pre-menopausal (26 ± 4:28 ± 7)	L 20 g/day (5)	One AE reported (U)	-	-	No effect
LOBO (2015)	DBRPCPT (T)	149 (74:75)	Post-menopausal (58 ± 5:58 ± 6)	M 1 g/day (365)	AE’s reported across study (S)	No difference between pre-and post-intervention	No difference between pre-and post-intervention	No effect
LYOO (2012)	DBRPCT (T)	52 (25:27)	Pre-menopausal (45.7 ± 12.7:47.5 ± 9.5)	M 3 g/day (7) then 5 g/day for (49)	AE’s reported across study (R)	No abnormal levels reported	No difference between pre-and post-intervention 3 showed mild increase in liver enzymes (2Cr,1Pl)	-
NEVES (2011)	DBRPCT (T)	26 (13:13)	Post-menopausal (59 ± 3:57 ± 3)	C 20 g/day (7) then 5 g/day (77)	No AE’s reported (R)	No difference between pre-and post-intervention	-	No effect
RAMIREZ-CAMPILLOᵆ (2016)	DBRPCT (E)	33 (10:10:10)	Pre-menopausal (23.1 ± 3.4:22.9 ± 1.7:22.5 ± 2.1)	C 20 g/day (7) then 5 g/day (35)	AE’s reported across study (S)	-	-	Effect
SILVA (1996)	DBRPCT (E)	16 (8:8)	Pre-menopausal (16.3 ± 1.8:15.7 ± 1.2)	L 20 g/day (21)	No AE’s reported (S)	-		No effect
THOMPSON (1996)	RPCT (E)	10 (−)	Pre-menopausal (university students)	M 2 g/day (42)	-	-	-	No effect
VANDENBERGHE (1997)	DBPCT (E)	19 (10:9)	Pre-menopausal (19–22)	C 20 g/day (4) then 5 g/day (73)	No AE’s reported (S)	No abnormal levels reported		Effect

Study Design: DBPCMT=Double blind placebo-controlled matched trial; DBPCT=Double blind placebo-controlled trial; DBRPCDRT =Double blind randomised placebo-controlled dose ranging trial; DBRPCMXT=Double blind randomised placebo-controlled matched crossover trial; DBRPCT=Double blind randomised placebo-controlled trial; DBRPCMT=Double blind randomised placebo-controlled matched trial; DBRPCPT=Double blind randomised placebo-controlled parallel trial; OLT=Open label trial; PSOL=Pilot study open label; RPCT=Randomised placebo-controlled trial; SBRPCMT=Single blind randomised placebo-controlled matched trial; SBRPCT=Single blind randomised placebo-controlled trial. Cr=Creatine group; Pl=Placebo group; E=Ergogenic; T=Therapeutic; M=maintenance dose only; C=Combination loading + maintenance dose, L=Loading dose only; AE=adverse event; Collection method, R=research initiated participant reporting; Collection method, S=participant self-reported; Collection method, U=unknown method of reporting; * 3 arms with different dosing regimens; ᵆ 3 arms (CrM, Placebo, Control); all=Mean and Standard deviation (SD) presented as total combined age group data.

## Supplementary Materials

The following are available online at https://www.mdpi.com/2072-6643/12/6/1780/s1, File S1: [A] Broad search strategy. Used for all databases (All creatine terms AND All creatine supplementation terms). [B] Narrow (refined) search strategy. Used for Ovid and Embase (with all adverse events (broad and narrow), excluding animals, and excluding creatine deficiencies) to validate against broad search strategy; File S2: [A] Number of adverse events reported by study. [B] Statements outlining generic reporting of adverse events by study. [C] Studies only reporting on change in body composition; File S3: Withdrawals, losses or cessation of intervention in females taking oral creatine monohydrate versus placebo.

## Short Running Head

Creatine supplementation in women; a safety review.

## Abbreviations

ATP	Adenosine triphosphate
CI	Confidence Interval
CrM	Creatine Monohydrate
I²	Statistical measure of heterogeneity
MD	Mean Difference
Pl	Placebo
RR	Risk Ratio
