# Supplementary material for: Risk of Adverse Outcomes in Females Taking Oral Creatine Monohydrate: A Systematic Review and Meta-Analysis
Source: Nutrients. 2020 Jun 15;12(6):1780. doi: 10.3390/nu12061780 (PMC7353222; doi:10.3390/nu12061780)
Supplement: Supplementary file 1 [file nutrients-12-01780-s001.zip › Nutrients. File S1_Search Strategy_Final (1).docx]

## **S1.**

**[A].** Broad search strategy used for all databases (All creatine AND All creatine supplementation terms).

1. exp Creatine/
2. ("total creatine" or "creatine phosphate" or "phosphocreatine" or "phosphorylcreatine").mp. [mp=title, abstract, original title, name of substance word, subject heading word, floating sub-heading word, keyword heading word, organism supplementary concept word, protocol supplementary concept word, rare disease supplementary concept word, unique identifier, synonyms]
3. 1 or 2
4. "creatine monohydrate".mp. [mp=title, abstract, original title, name of substance word, subject heading word, floating sub-heading word, keyword heading word, organism supplementary concept word, protocol supplementary concept word, rare disease supplementary concept word, unique identifier, synonyms]
5. (oral adj3 creatine).mp. [mp=title, abstract, original title, name of substance word, subject heading word, floating sub-heading word, keyword heading word, organism supplementary concept word, protocol supplementary concept word, rare disease supplementary concept word, unique identifier, synonyms]
6. (creatine adj3 diet*).mp. [mp=title, abstract, original title, name of substance word, subject heading word, floating sub-heading word, keyword heading word, organism supplementary concept word, protocol supplementary concept word, rare disease supplementary concept word, unique identifier, synonyms]
7. (creatine adj3 nutraceutical).mp. [mp=title, abstract, original title, name of substance word, subject heading word, floating sub-heading word, keyword heading word, organism supplementary concept word, protocol supplementary concept word, rare disease supplementary concept word, unique identifier, synonyms]
8. (creatine adj2 monoh*).mp. [mp=title, abstract, original title, name of substance word, subject heading word, floating sub-heading word, keyword heading word, organism supplementary concept word, protocol supplementary concept word, rare disease supplementary concept word, unique identifier, synonyms]
9. (supp* adj3 creatine).mp. [mp=title, abstract, original title, name of substance word, subject heading word, floating sub-heading word, keyword heading word, organism supplementary concept word, protocol supplementary concept word, rare disease supplementary concept word, unique identifier, synonyms]
10. 4 or 5 or 6 or 7 or 8 or 9
11. 3 and 10

## **[B].** Narrow (refined) search strategy used for Ovid and Embase ((with all adverse events (broad and narrow), excluding animals, and excluding creatine deficiencies)).

1. (adverse event* or adverse reaction* or adverse effect*).mp. [mp=title, abstract, original title, name of substance word, subject heading word, floating sub-heading word, keyword heading word, organism supplementary concept word, protocol supplementary concept word, rare disease supplementary concept word, unique identifier, synonyms]
2. exp Creatine/ad, ae, de, po, tu, to, ur [Administration & Dosage, Adverse Effects, Drug Effects, Poisoning, Therapeutic Use, Toxicity, Urine]
3. 12 or 13
4. exp Dietary Supplements/ae, po, to [Adverse Effects, Poisoning, Toxicity]
5. ("drug related side effects" or "drug related adverse reactions").mp. [mp=title, abstract, original title, name of substance word, subject heading word, floating sub-heading word, keyword heading word, organism supplementary concept word, protocol supplementary concept word, rare disease supplementary concept word, unique identifier, synonyms]
6. ("side effect*" or "negative effect" or toxicity or safety or "negative outcome").mp. [mp=title, abstract, original title, name of substance word, subject heading word, floating sub-heading word, keyword heading word, organism supplementary concept word, protocol supplementary concept word, rare disease supplementary concept word, unique identifier, synonyms]
7. (death or mortality or morbidity).mp. [mp=title, abstract, original title, name of substance word, subject heading word, floating sub-heading word, keyword heading word, organism supplementary concept word, protocol supplementary concept word, rare disease supplementary concept word, unique identifier, synonyms]
8. 12 or 13 or 14 or 15 or 16 or 17 or 18
9. (creatine and nephropathy).mp. [mp=title, abstract, original title, name of substance word, subject heading word, floating sub-heading word, keyword heading word, organism supplementary concept word, protocol supplementary concept word, rare disease supplementary concept word, unique identifier, synonyms
10. (creatine and "heterocyclic amines").mp. [mp=title, abstract, original title, name of substance word, subject heading word, floating sub-heading word, keyword heading word, organism supplementary concept word, protocol supplementary concept word, rare disease supplementary concept word, unique identifier, synonyms]
11. ((creatine and "muscle weakness") or "muscle fatigue" or "muscle rupture" or "muscle cramps").mp. [mp=title, abstract, original title, name of substance word, subject heading word, floating sub-heading word, keyword heading word, organism supplementary concept word, protocol supplementary concept word, rare disease supplementary concept word, unique identifier, synonyms]
12. (creatine and bloat*).mp. [mp=title, abstract, original title, name of substance word, subject heading word, floating sub-heading word, keyword heading word, organism supplementary concept word, protocol supplementary concept word, rare disease supplementary concept word, unique identifier, synonyms]
13. (creatine and cancer).mp. [mp=title, abstract, original title, name of substance word, subject heading word, floating sub-heading word, keyword heading word, organism supplementary concept word, protocol supplementary concept word, rare disease supplementary concept word, unique identifier, synonyms]
14. (creatine and nausea).mp. [mp=title, abstract, original title, name of substance word, subject heading word, floating sub-heading word, keyword heading word, organism supplementary concept word, protocol supplementary concept word, rare disease supplementary concept word, unique identifier, synonyms]
15. (creatine and vomiting).mp. [mp=title, abstract, original title, name of substance word, subject heading word, floating sub-heading word, keyword heading word, organism supplementary concept word, protocol supplementary concept word, rare disease supplementary concept word, unique identifier, synonyms]
16. (creatine and gastroint*).mp. [mp=title, abstract, original title, name of substance word, subject heading word, floating sub-heading word, keyword heading word, organism supplementary concept word, protocol supplementary concept word, rare disease supplementary concept word, unique identifier, synonyms]
17. (creatine and heat).mp. [mp=title, abstract, original title, name of substance word, subject heading word, floating sub-heading word, keyword heading word, organism supplementary concept word, protocol supplementary concept word, rare disease supplementary concept word, unique identifier, synonyms]
18. (creatine and hepat*).mp. [mp=title, abstract, original title, name of substance word, subject heading word, floating sub-heading word, keyword heading word, organism supplementary concept word, protocol supplementary concept word, rare disease supplementary concept word, unique identifier, synonyms]
19. (creatine and weight).mp. [mp=title, abstract, original title, name of substance word, subject heading word, floating sub-heading word, keyword heading word, organism supplementary concept word, protocol supplementary concept word, rare disease supplementary concept word, unique identifier, synonyms]
20. (creatine and "body mass").mp. [mp=title, abstract, original title, name of substance word, subject heading word, floating sub-heading word, keyword heading word, organism supplementary concept word, protocol supplementary concept word, rare disease supplementary concept word, unique identifier, synonyms]
21. (creatine and "weight gain").mp. [mp=title, abstract, original title, name of substance word, subject heading word, floating sub-heading word, keyword heading word, organism supplementary concept word, protocol supplementary concept word, rare disease supplementary concept word, unique identifier, synonyms]
22. (creatine and "fat free mass").mp. [mp=title, abstract, original title, name of substance word, subject heading word, floating sub-heading word, keyword heading word, organism supplementary concept word, protocol supplementary concept word, rare disease supplementary concept word, unique identifier, synonyms]
23. (creatine and renal*).mp. [mp=title, abstract, original title, name of substance word, subject heading word, floating sub-heading word, keyword heading word, organism supplementary concept word, protocol supplementary concept word, rare disease supplementary concept word, unique identifier, synonyms]
24. ((creatine and kidney) or renal or "renal function" or "renal disease" or nephropathy).mp. [mp=title, abstract, original title, name of substance word, subject heading word, floating sub-heading word, keyword heading word, organism supplementary concept word, protocol supplementary concept word, rare disease supplementary concept word, unique identifier, synonyms]
25. (creatine and dehydrat*).mp. [mp=title, abstract, original title, name of substance word, subject heading word, floating sub-heading word, keyword heading word, organism supplementary concept word, protocol supplementary concept word, rare disease supplementary concept word, unique identifier, synonyms]
26. (creatine and thirst).mp. [mp=title, abstract, original title, name of substance word, subject heading word, floating sub-heading word, keyword heading word, organism supplementary concept word, protocol supplementary concept word, rare disease supplementary concept word, unique identifier, synonyms]
27. 20 or 34 or 35
28. 20 or 21 or 22 or 23 or 24 or 25 or 26 or 27 or 28 or 29 or 30 or 31 or 32 or 33 or 34 or 36 or 37 or 38
29. 11 and 19
30. 11 and 39
31. 41 not 40
32. from 42 keep 3,5,7-10,12-13,27,29,33,37,44,46,49-51,53-55,57,60-61,66-69,71,77-80,83-89,91,94-96,100-103,105,109-110,113
33. 40 or 43
34. exp animals/ not humans/
35. 44 not 45
36. ("creatine deficiency" or "creat* deficit*" or "mitochond* disease" or "mitoch* deficit*").mp. [mp=title, abstract, original title, name of substance word, subject heading word, floating sub-heading word, keyword heading word, organism supplementary concept word, protocol supplementary concept word, rare disease supplementary concept word, unique identifier, synonyms]
37. 46 not 47
38. (deficiency or deficit).mp. [mp=title, abstract, original title, name of substance word, subject heading word, floating sub-heading word, keyword heading word, organism supplementary concept word, protocol supplementary concept word, rare disease supplementary concept word, unique identifier, synonyms]
39. 47 or 49
40. 46 not 50
41. 46 not 51
42. from 52 keep 6-7,14,17,28,38-39,41-42,44-46,52-53
43. 51 or 53
