# Supplementary material for: Risk of Adverse Outcomes in Females Taking Oral Creatine Monohydrate: A Systematic Review and Meta-Analysis
Source: Nutrients. 2020 Jun 15;12(6):1780. doi: 10.3390/nu12061780 (PMC7353222; doi:10.3390/nu12061780)
Supplement: Supplementary file 1 [file nutrients-12-01780-s001.zip › Nutrients. File S2_Outcomes across studies_Final (1).docx]

**S2**

**[A]** Number of adverse events reported by study

| ADVERSE EVENTS (Symptoms) | Author | Study Design | Creatine Monohydrate | Placebo |
| --- | --- | --- | --- | --- |
| Nausea OR  vomiting | Hellem | OLT | 1 | NA |
|  | Kondo^2011^ | OLT | 1 | NA |
|  | ᵃLyoo | DBRPCT | 9 | 6 |
|  | Chilibeck | DBRPCPT | 1 | 1 |
|  | Larson-Meyer | DBRCT | 0 | 1 |
| GIT discomfort | Hellem | OLT | 3 | NA |
|  | Hamilton | RPCT | 1 | 1 |
|  | Kondo^2016^ | DBRPCDRT | 11 | 4 |
|  | Lobo | DBRPCPT | 1 | 2 |
|  | Ramirez | DBRCT | 1 | 0 |
|  | Cox | DBPCTMX | 1 | 0 |
| Diarrhoea | Hellem | OLT | 4 | NA |
|  | Chilibeck | DBRPCPT | 1 | 0 |
| Constipation | Lyoo | DBRPCT | 2 | 0 |
|  | Chilibeck | DBRPCPT | 1 | 0 |
| Irritable bowel | Chilibeck | DBRPCPT | 1 | 0 |
| Indigestion OR  dyspepsia OR  bloated OR  decreased appetite | Hellem | OLT | 1 | NA |
|  | Kondo^2011^ | OLT | 1 | NA |
|  | Lyoo | DBRPCT | 1 | 2 |
|  | Chilibeck | DBRPCPT | 1 | 0 |
| Sense of weight gain | Larson-Meyer | DBRCT | 0 | 2 |
| Headache OR  tension headache | Hellem | OLT | 3 | NA |
|  | Kondo^2011^ | OLT | 3 | NA |
|  | ᵃLyoo | DBRPCT | 6 | 13 |
| Blurred vision | ∞Hellem | OLT | 1 | NA |
| Increased dreaming | Lyoo | DBRPCT | 0 | 1 |
| Increased sweating | Lyoo | DBRPCT | 2 | 2 |
| Muscle cramps | Hellem | OLT | 2 | NA |
|  | Chilibeck | DBRPCPT | 2 | 0 |
| Flank pain | ∞Hellem | OLT | 1 | NA |
| Lightheaded OR  dizziness | ∞Hellem | OLT | 1 | NA |
|  | Lyoo | DBRPCT | 5 | 3 |
| Difficulty urinating | Lyoo | DBRPCT | 1 | 1 |
| Increased thirst OR  dry mouth | Lyoo | DBRPCT | 1 | 0 |
|  | ∞Hellem | OLT | 1 | NA |
| Tingling or numbness in hands | ∞Hellem | OLT | 1 | NA |
| Swelling in hands | ∞Hellem | OLT | 4 | NA |
| Cold/flu symptoms | ∞Hellem | OLT | 10 | NA |
| Agitation OR  restlessness | Lyoo | DBRPCT | 2 | 5 |
| Difficulty concentrating | Lyoo | DBRPCT | 1 | 3 |
| Palpitations | Lyoo | DBRPCT | 0 | 1 |

Study Design: OLT=Open label trial; DBRPCT=Double blind randomised placebo-controlled trial; DBRPCPT =Double blind randomised placebo-controlled parallel arm trial; RPCT=Randomised placebo-controlled trial; DBRPCDRT =Double blind randomised placebo-controlled dose ranging trial; DBPCTMX=Double blind placebo-controlled matched trial; NA=Not applicable, no control group; ᵃ Lyoo reported tension headache and nausea/vomiting as symptoms more likely related to SSRI than creatine. ∞Events deemed unlikely to be related to study medication.

Symptoms reported in manuscript but not included in above table were deemed to be either pre-existing or not related to study intervention by the authors. Kondo ^2011^ reported; tremor (n=1), suicidal ideation (n=3), cold/flu symptoms (n=4), bruising (n=1), nosebleed (n=1), acne (n=1). Larson-Meyer stated 10 of 12 participants reported increased urination, but the author deemed this attributable to increased fluid consumption [300-500mls] with supplement. Lyoo reported sleeping difficulties, as either insomnia or somnolence across both groups (CrM,6:Pl,8).

**[B]** Statements outlining generic reporting of adverse events by study

| **Author** | **Study Design** | **SYMPTOMS (Reported within manuscript)** |
| --- | --- | --- |
| Aguiar | DBRPCT | No adverse events were reported by the participants. |
| Alves | DBRPCPT | There were no self-reported side effects throughout the trial. Moreover, no adverse events were reported through the trial. |
| Ayoama | DBRPCPT | No significant change in serum GOT, GPT or blood urea but significant rise in serum creatine in CrM groups compared to placebo. |
| Benton | DBRPCT | 85%Pl reported no side effects, 74%Cr reported no side effects. The side effects were in all cases minor, for example reporting feeling bloated or having a headache. |
| Brenner | DBRPCT | Two subjects were dismissed from the study due to illness. Another subject became injured (compartment syndrome). |
| Canete | SBRPCT | There were no concomitant adverse effects such as muscle cramps, gastrointestinal complaints or changes in circulating levels of liver enzymes. |
| Ferguson | DBRPCMX | No spontaneous or unusual side effects were reported during the entire duration of the study. |
| Gotshalk | DBPCTMX | After the 7 days subjects were administered a 10-point Likert scale exit questionnaire to record onset of muscle cramps, gastrointestinal distress, and to assess physical well-being. There were no significant differences between the CR group, the PL group in blood pressure and the subjective Likert scores for gastrointestinal distress and muscle cramps. No adverse side-effects were reported by either group. |
| Gualano | DBRPCPT | There were no self-reported side effects throughout the study. Clinical examinations did not reveal adverse events potentially associated with creatine supplementation or resistance training. |
| Kambis | DBPCTMX | No complaints of side effects in either group. |
| Leader | OLT | No adverse effects or serious adverse events reported during trial. |
| Ledford | DBRPCT | One subject became ill during repetitive Wingate testing following both supplementation regimens and failed to complete the testing protocol. |
| Neves | DBRPCT | No adverse effects were reported. |
| Silva | DBRPCT | No side effects were reported during the entire duration of the study. |
| Vandenberghe | DBPCT | No side effects reported |

DBPCTMX=Double blind placebo controlled matched trial; DBRPCT=Double-blind randomised placebo-controlled trial; DBRPCT=Double-blind randomised placebo-controlled trial; OLT=Open label trial; DBRPCMX=Double blind randomised placebo controlled matched trial, DBRPCXT=Double blind randomised placebo-controlled crossover trial, RPCT=randomised placebo-controlled trial; GOT/AST=aspartate aminotransferase; GPT/ALT=alanine aminotransferase

**[C]**  Studies only reporting on change in body composition

| **Author** | **Study Design** | **SYMPTOMS** |
| --- | --- | --- |
| Atakan | DBRPCT | Body composition only |
| Eckerson | DBRPCMXT | Body composition only |
| Forbes | DBRPCT | Body composition only |
| Thompson | RPCT | Body composition only |

DBRPCT=Double blind randomised placebo-controlled trial; DBRPCMXT=Double blind randomised placebo-controlled matched crossover trial; DBRPCT=Double blind randomised placebo-controlled parallel arm trial; RPCT=Randomised placebo-controlled trial.
