# Supplementary material for: Risk of Adverse Outcomes in Females Taking Oral Creatine Monohydrate: A Systematic Review and Meta-Analysis
Source: Nutrients. 2020 Jun 15;12(6):1780. doi: 10.3390/nu12061780 (PMC7353222; doi:10.3390/nu12061780)
Supplement: Supplementary file 1 [file nutrients-12-01780-s001.zip › Nutrients. File S3. AttritionTable_Final(1).docx]

# S3. Withdrawals, losses, or cessation of intervention in females taking oral creatine monohydrate versus placebo.

| Author  (Classification of study) | n participants  (GRoup allocation) | % of studies  overall participants | Timing | Group ALLOCATION, Reason-if reported |
| --- | --- | --- | --- | --- |
| alves (T) | 4 (1CrM, 3Pl) | 12.5 | Post randomisation | Both groups-for personal reasons. |
| benton (T) * | 7 (unknown) | 5 | Unknown | 128 identified in abstract but 121 reported in manuscript. |
| Brenner (E) | 4 (3CrM,1Pl) | 20 | Post randomisation | CrM group: 1-Compartment syndrome, 2-illness,  Pl group: 1-left team. |
| chilibeck (T) | 16 (10CrM,6Pl) | 34 | Post randomisation | CrM group: 5-time commitment, 2-personal health issues, 1-irritable bowel, 1-diarrhoea, 1-did not like supplement.  Pl group: 3-time commitment, 1-work injury, 1-low creatinine clearance, 1- commenced hormone replacement therapy. All discontinued intervention. |
| Cox (E) * | 2 (unknown) | 14 | Pre randomisation | Group unknown: 2 left; 1- injury, 1-illness. Appeared to be prior to intervention allocation. |
| forbes (E) | 1 (0CrM,1Pl) | 5 | Unknown | Pl group: no reason given |
| gualano (T) | 14 (7CrM,7Pl) | 19 | Post randomisation | Both groups-personal reasons. |
| hamilton (E) | 4 (3CrM,1Pl) | 14 | Post randomisation | Both groups-personal reasons. |
| hellem (T) * | 3 (CrM All) | 20 | Post treatment commencement | CrM: 1-time commitment, 1-admission to hospital, 1-loss to follow up.  Author reported-‘No participant withdrew due to adverse events.’ |
| Kondo ^2016^ (T) | 5 (3CrM,2Pl) | 15 | Post randomisation | Author reported, ‘No subject withdrew from the study due to CrM-associated adverse events.’ |
| Larson-meyer (E) | 3 (0CrM,3 Pl) | 21 | Post randomisation | Pl group: 3 ceased intervention only because of GIT symptoms but remained in study. |
| leader (T) * | 14 (CrM All) | 47 | Post treatment commencement | CrM: 13 of 14 dropped out after first meeting; 1 dropped out after 2nd meeting (9-were loss to follow up, 5-were treatment noncompliant). |
| ledford (E) * | 1 (NA) | 1 | Post randomisation | 1 subject fell ill after both supplementation regimens. |
| Lobo (T) ᵓ | 40 (18CrM, 22Pl) | 27 | Post randomisation | CrM group: 15-personal reasons, 2-non trial related medical reasons,1-GIT symptoms.  Pl group: 15-personal reasons, 6-non trial related medical reasons, 2-GIT symptoms. |
| Lyoo (T) ˠ | 13 (8CrM,5Pl) | 25 | Post randomisation | CrM group: 2-withdrew consent, 4-loss to follow up, 2-adverse event.  Pl group: 2-withdrew consent, 2-loss to follow up, 1-adverse event. |
| neves (T) | 2 (0CrM,2Pl) | 8 | Post randomisation | Pl group: personal reasons |

T=THERAPEUTIC, E=ERGOGENIC. CrM=Creatine monohydrate, Pl=Placebo group. *Studies not included in total attrition numbers reported across groups, ᵓ Non trial related events included hepatitis, knee surgery, onset of diabetes, ankle or knee sprains, and diverticulitis. ˠ Reported by author; no statistically significant difference between losses across groups, (df=1, p=0.26).
